# A Difficult Case of Cardio-Cerebral Infarction Syndrome With Left Ventricular Thrombus

**DOI:** 10.7759/cureus.60196

**Published:** 2024-05-13

**Authors:** Xiong Khee Cheong, Juen Kiem Tan, Zarrin Kang, Najma Kori, Petrick Ramesh Periyasamy

**Affiliations:** 1 Internal Medicine, Universiti Kebangsaan Malaysia Medical Centre, Kuala Lumpur, MYS; 2 Cardiology, Hospital Raja Permaisuri Bainun Ipoh, Ipoh, MYS

**Keywords:** cardioembolic stroke, antiplatelets, anticoagulant therapy, ventricular thrombus, myocardial infarction

## Abstract

Left ventricular thrombus is a major complication following myocardial infarction, particularly in patients with anterior myocardial infarction or dilated cardiomyopathies regardless of coronary reperfusion therapy. Embolization of mural thrombus is one of the major causes of large vessel occlusion ischemic stroke. A combination therapy of antiplatelet (single or dual antiplatelet) and anticoagulant is mandatory in the management of myocardial infarction and left ventricular thrombus with or without stroke. To our knowledge, there are no guidelines on the optimal regimen (dual or triple therapies) and timing of administration in cases of cardio-cerebral infarction. It is difficult for clinicians to balance the risks of intracranial hemorrhage and coronary stent thrombosis. Here, we describe the case of a gentleman who had recently undergone coronary intervention and presented with ischemic stroke and left ventricular thrombus, along with the management challenges in this scenario.

## Introduction

Cardioembolic stroke and myocardial infarction (MI) cause significant morbidity and mortality worldwide. However, following the implementation of coronary reperfusion therapy with primary percutaneous coronary intervention (PCI) and thrombolysis therapy, the incidence rate of left ventricular (LV) thrombus post-MI has decreased dramatically. LV thrombus is a major complication following MI, particularly in patients with anterior MI or dilated cardiomyopathies regardless of coronary reperfusion therapy. Systemic embolization is one of the major causes of large vessel occlusion ischemic stroke [1,2]. A combination therapy of antiplatelet (single or dual antiplatelet) and anticoagulant is mandatory in the management of MI and LV thrombus with or without stroke. To our knowledge, there are no guidelines on the optimal regimen (dual or triple therapies) and timing of administration in cases of cardio-cerebral infarction. It is difficult for clinicians to balance the risks of intracranial hemorrhage and stent thrombosis. We present the case of a gentleman who had recently undergone coronary intervention and presented with ischemic stroke and LV thrombus, as well as the management challenges in this scenario.

## Case presentation

**Figure 1:**
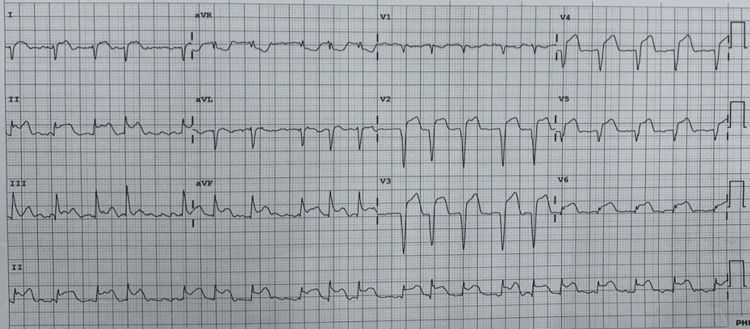
Electrocardiogram showed ST elevation with Q wave in V2-5.

**Figure 2:**
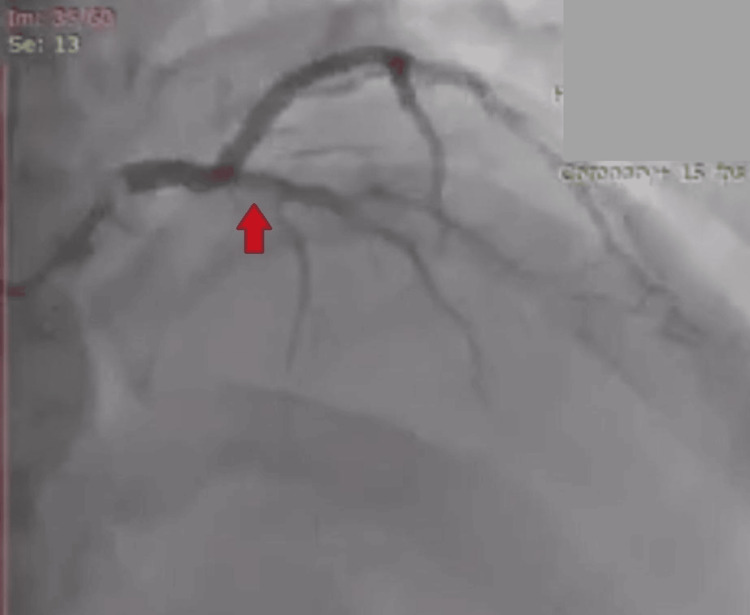
Coronary angiogram showed left anterior descending artery occlusion (red arrow).

A 39-year-old social smoker gentleman presented to the emergency department with a five-hour history of central chest pain. His vital signs were stable upon arrival, with a blood pressure of 136/86 mmHg and a pulse rate of 90 beats per minute. A cardiovascular examination revealed normal heart sounds and no additional murmurs. An examination of other systems was unremarkable. Electrocardiogram showed ST elevation with Q wave at lead V2-5 (Figure 1). Troponin T was elevated. A diagnosis of acute anterior MI Killip 1 was made. He was given intravenous thrombolysis therapy as PCI was unavailable in that center. He proceeded for PCI after the failure of thrombolysis. Coronary angiography revealed occlusion of the proximal left anterior descending artery (Figure 2). As a result, revascularization therapy with PCI and placement of a drug-eluting stent was performed. The echocardiogram showed a 45% ejection fraction, hypokinesia at the anterior and apical regions, and no intracardiac thrombus. He was discharged home with dual antiplatelets four days after PCI.

He returned to the emergency room two days later with left-sided body weakness and expressive aphasia. On examination, the Glasgow Coma Scale score was E4V2M6, blood pressure was 170/89 mmHg, and heart rate was 80 beats per minute. A neurological examination revealed left hemiparesis with muscle power of 2/5 and a National Institutes of Health Stroke Scale score of 15.

**Figure 3:**
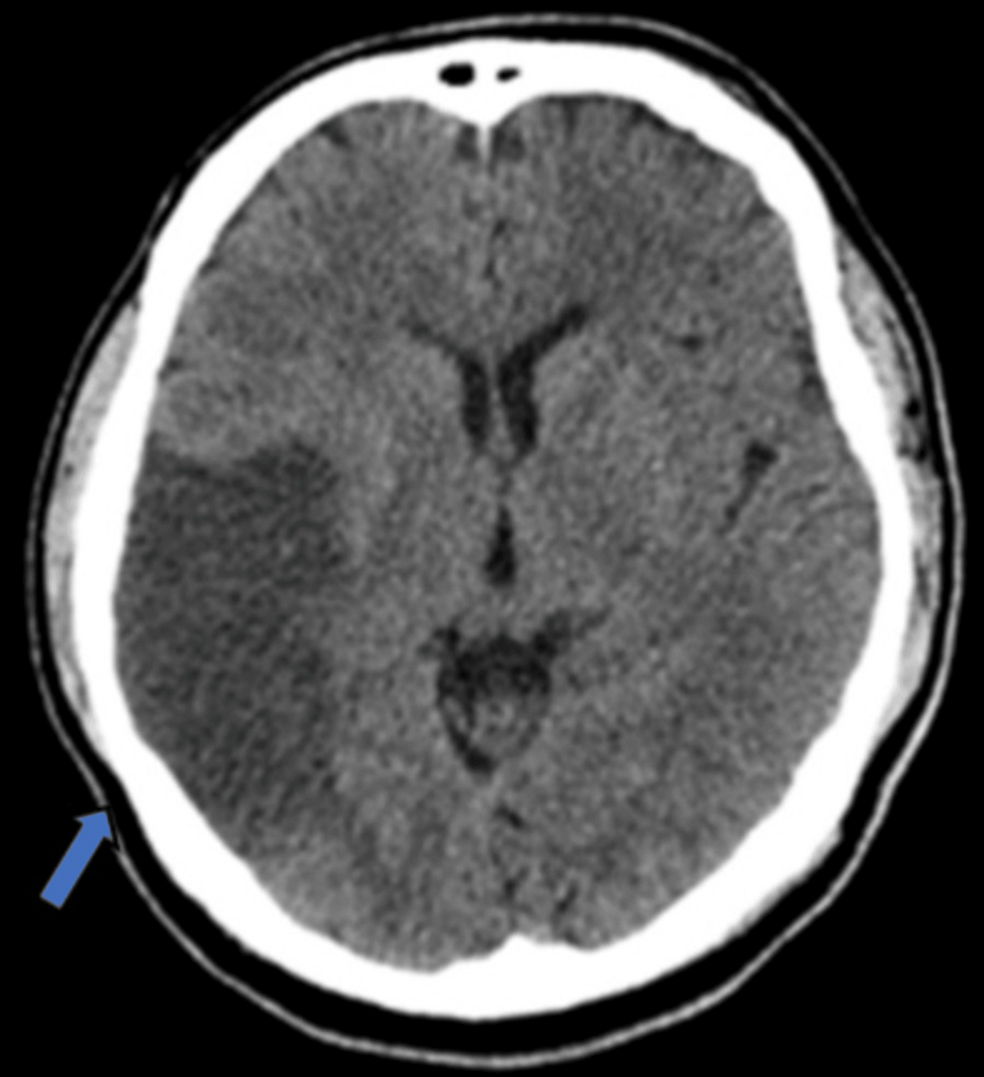
Axial non-contrast CT of the brain showing a large infarct (blue arrow) in the right middle cerebral artery territory with sulci effacement.

**Figure 4:**
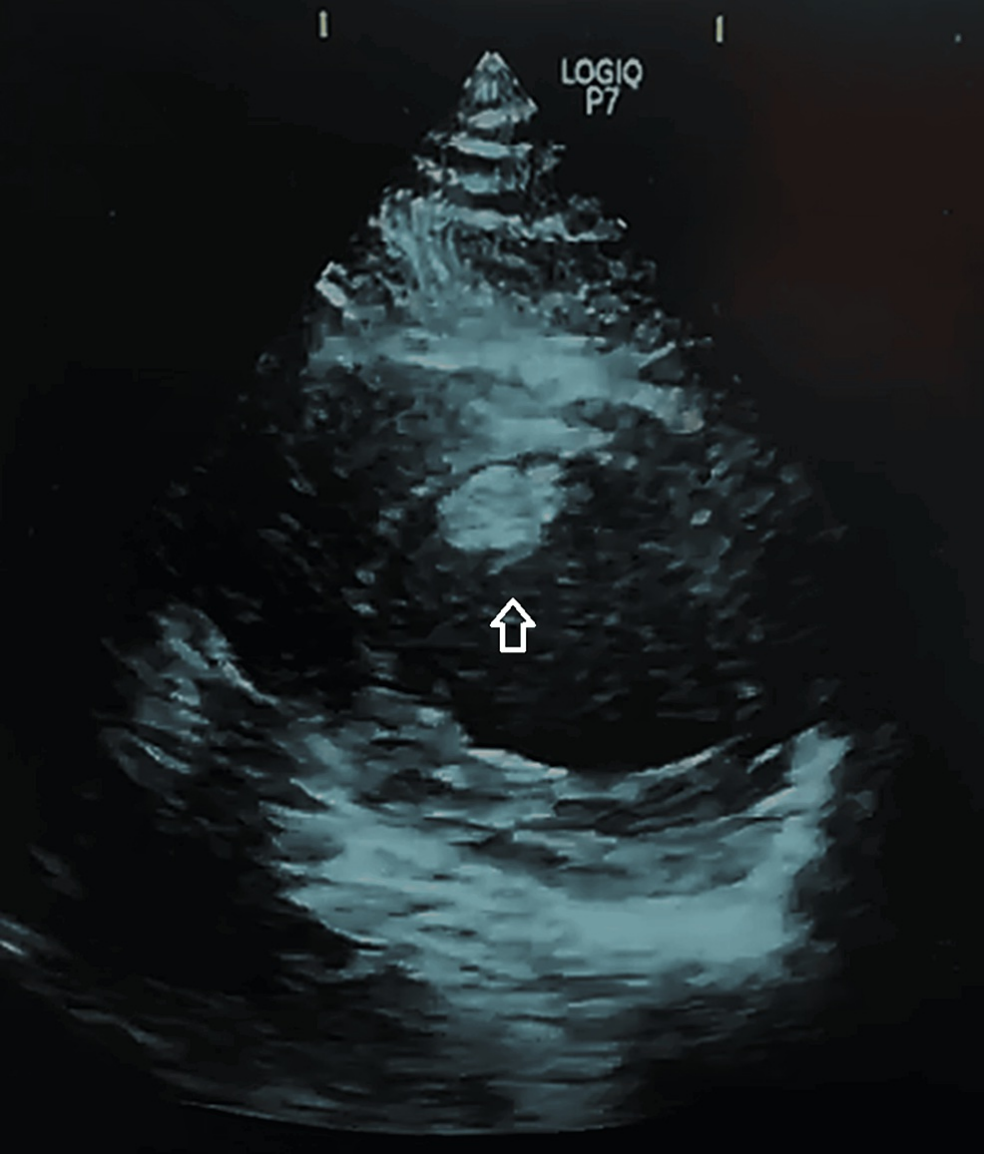
Parasternal short-axis view at the level of papillary muscles showing a large apical left ventricle thrombus (white arrow).

A computed tomography (CT) of the brain revealed a large right middle cerebral artery territory infarction (Figure 3). He did not receive thrombolysis or mechanical thrombectomy because it was beyond the therapeutic window period. A repeated echocardiogram showed an LV apical thrombus (1.5 × 0.9 cm) (Figure 4). A 48-hour follow-up CT of the brain revealed no intracranial hemorrhagic transformation. Blood investigation revealed a normal hemoglobin of 14.5 g/dL with a normal platelet count of 410 × 10^9^. Renal and liver functions were normal. Following a multidisciplinary team discussion, subcutaneous heparin with a single antiplatelet (clopidogrel) was initiated due to the risk of intracranial hemorrhagic transformation. On the fifth post-event day, aspirin was restarted, and the anticoagulant was changed to warfarin before discharge. During his hospitalization, he received regular neurological rehabilitation and speech therapy. Upon discharge, the patient demonstrated good neurological recovery and was able to ambulate with a walking frame. He regained full neurological recovery one month after the event. A follow-up echocardiogram revealed no residual LV thrombus and an improved LV ejection fraction of 52%.

## Discussion

Cardioembolic stroke is a fatal complication following systemic embolization of LV thrombus in the post-MI population. Following an acute MI, the incidence of LV thrombus ranges from 2.7% to 9.1%, while ischemic stroke ranges from 0.7% to 2.2% [3-6]. Despite tremendous improvement in coronary revascularization with percutaneous intervention therapy, there is still up to 15% risk of LV thrombus formation in the first three months in acute MI patients treated by PCI, particularly in those with poor LV ejection fraction [7,8]. The mechanisms of LV thrombus formation can be explained by Virchow’s triad, which is blood pooling within cardiac chambers following MI, in addition to the release of inflammatory cells and endocardial injury which further contribute to the formation of LV thrombus [1].

In cases of cardioembolic stroke, a transthoracic echocardiogram is useful to detect LV thrombus and cardiac pathology such as patent foramen ovale. The main advantage of this modality is that it is easily accessible and can be used as a bedside diagnostic tool. Other modalities such as cardiac magnetic resonance imaging or transesophageal echocardiography provide a detailed assessment of cardiomyopathies and valvular abnormalities; however, they are not widely available.

Ischemic stroke following a recent MI poses a therapeutic challenge for clinicians. It is difficult to strike a balance between an efficacious management of post-PCI MI with thromboembolic stroke while balancing the risk of intracranial hemorrhagic transformation. The management is more challenging in the event of a concurrent LV thrombus [3,7].

American and European guidelines recommend triple therapy for the management of acute MI with LV thrombus which consists of dual antiplatelet and anticoagulant primarily oral vitamin K antagonists for three to six months with serial echocardiography assessment [1,9,10]. There is still a lack of consensus on the optimal antithrombotic regimen for MI and LV thrombus with concurrent ischemic stroke. Meta-analyses have shown a controversial result on the use of direct oral anticoagulants (DOACs) compared to vitamin K antagonists for the treatment of LV thrombus [5,11,12]. Randomized studies are needed to assess DOAC efficacy, especially in large thrombus burden populations with concurrent ischemic stroke.

We adopted a stepwise approach in this case, starting with dual therapy (clopidogrel and warfarin) and adding aspirin later. This allowed us to closely monitor the patient’s neurological progress with hemorrhagic risk while balancing the need for anticoagulants for LV thrombus management and antiplatelets after stent implantation. This case emphasizes the difficulty and challenges of managing cardioembolic stroke in a post-MI patient with LV thrombus. The antithrombotic regimen and duration should be tailored to the patient’s clinical condition and the risk of ischemic and bleeding events. As a result, a multidisciplinary team approach is required because there are currently no clear guidelines on the optimal timing for restarting antithrombotic agents following an event.

## Conclusions

This case illustrates the difficulties that clinicians encounter while balancing the risks of cerebral hemorrhage with stent thrombosis. In the acute setting of a cardio-cerebral infarction with LV thrombus, the timing and duration of antiplatelet and anticoagulant therapy should be individualized.
